# Inflammation Markers in Multiple Sclerosis: CXCL16 Reflects and May Also Predict Disease Activity

**DOI:** 10.1371/journal.pone.0075021

**Published:** 2013-09-19

**Authors:** Trygve Holmøy, Kristin Ingeleiv Løken-Amsrud, Søren Jacob Bakke, Antonie G. Beiske, Kristian S. Bjerve, Harald Hovdal, Finn Lilleås, Rune Midgard, Tom Pedersen, Jutrate Šaltytė Benth, Øivind Torkildsen, Stig Wergeland, Kjell-Morten Myhr, Annika E. Michelsen, Pål Aukrust, Thor Ueland

**Affiliations:** 1 Department of Neurology, Akershus University Hospital, Lørenskog, Norway; 2 Institute of Clinical Medicine, University of Oslo, Oslo, Norway; 3 Department of Neurology, Innlandet Hospital Trust, Lillehammer, Norway; 4 Department of Neuroradiology, Oslo University Hospital, Oslo, Norway; 5 Multiple Sclerosis Centre Hakadal, Hakadal, Norway; 6 Department of Medical Biochemistry, St. Olavs Hospital, Trondheim University Hospital, Trondheim, Norway; 7 Department of Neurology, St. Olavs Hospital, Trondheim University Hospital, Trondheim, Norway; 8 Curato Oslo, Oslo, Norway; 9 Department of Neurology, Molde Hospital, Molde, Norway; 10 Unit for Applied Clinical Research, Norwegian University of Science and Technology, Trondheim, Norway; 11 Unilabs Drammen, Drammen, Norway; 12 Helse Sør-Øst Health Services Research Centre, Akershus University Hospital, Lørenskog, Norway; 13 Norwegian Multiple Sclerosis Competence Centre, Department of Neurology, Haukeland University Hospital, Bergen, Norway; 14 KG KG Jebsen MS Research Centre, Department of Clinical Medicine, University of Bergen, Bergen, Norway; 15 Research Institute of Internal Medicine, Oslo University Hospital Rikshospitalet, Oslo, Norway; Institute Biomedical Research August Pi Sunyer (IDIBAPS) - Hospital Clinic of Barcelona, Spain

## Abstract

**Background:**

Serum markers of inflammation are candidate biomarkers in multiple sclerosis (MS). ω-3 fatty acids are suggested to have anti-inflammatory properties that might be beneficial in MS. We aimed to explore the relationship between serum levels of inflammation markers and MRI activity in patients with relapsing remitting MS, as well as the effect of ω-3 fatty acids on these markers.

**Methods:**

We performed a prospective cohort study in 85 relapsing remitting MS patients who participated in a randomized clinical trial of ω-3 fatty acids versus placebo (the OFAMS study). During a period of 24 months 12 repeated magnetic resonance imaging (MRI) scans and nine serum samples were obtained. We measured 10 inflammation markers, including general down-stream markers of inflammation, specific markers of up-stream inflammatory pathways, endothelial action, and matrix regulation.

**Results:**

After Bonferroni correction, increasing serum levels of CXCL16 and osteoprotegerin were associated with low odds ratio for simultaneous MRI activity, whereas a positive association was observed for matrix metalloproteinase (MMP) 9. CXCL16 were also associated with low MRI activity the next month, but this was not significant after Bonferroni correction. In agreement with previously reported MRI and clinical results, ω-3 fatty acid treatment did not induce any change in the inflammation markers.

**Conclusions:**

Serum levels of CXCL16, MMP-9, and osteoprotegerin reflect disease activity in MS, but are not affected by ω-3 fatty acid treatment. CXCL16 could be a novel biomarker and potential predictor of disease activity in MS.

## Introduction

The inflammatory response in multiple sclerosis (MS) involves a complex interaction between T cells, B cells, macrophages, dendritic cells, and endothelial cells. Whereas several inflammatory cytokines and growth factors have been suggested as potential mediators [1], their regulation and relative importance are not clear. Moreover, the immunopathogenesis of MS also involves matrix remodeling, including activation of various matrix degrading enzymes such as matrix metalloproteinases (MMPs) [2]. In addition, inflammatory mediators could also mediate neuroprotection and repair [3,4].

Current MS treatments have limited effect and complementary treatments, including ω-3 fatty acids (FA), are used by many patients [5]. However, although ω-3 FA are suggested to have anti-inflammatory properties [6–8], the evidence underpinning their ability to modulate inflammation in MS is largely unknown. We have recently reported that the ω-3 FA in Multiple Sclerosis (OFAMS) study, a randomized clinical trial of 1350 mg eicosapentaenoic acid (EPA) and 850 mg docosahexaenoic acid (DHA) daily versus placebo (corn oil), failed to show any effect on disease activity as assessed by magnetic resonance imaging (MRI) [9]. Whether this reflects an inability to modulate inflammation was not examined in the primary report.

The aim of this sub-study was to assess (i) the association between inflammatory markers and simultaneous or subsequent MRI activity in the OFAMS study population, and (ii) whether treatment with ω-3 fatty acids affects serum markers of inflammation. We examined general down-stream markers of inflammation including pentraxin 3 (PTX3), osteopontin (OPN), soluble tumor necrosis factor receptor type 1 (sTNF-R1), osteoprotegerin (OPG) and interleukin (IL)-1 receptor antagonist (IL-1RA), more specific markers of up-stream inflammatory pathways such as CXCL16, CCL21 and transforming growth factor (TGF) β, markers of endothelial action including activated leukocyte cell adhesion molecule (ALCAM) and MMP-9. All together, these markers will reflect inflammatory pathways (e.g., PTX3, OPG, sTNF-R1, IL-1Ra and CXCL16), the interaction between leukocytes and endothelial cells (e.g., PTX3, ALCAM, CXCL16 and CCL21) and extracellular matrix remodeling (e.g., OPN, OPG, TGFβ and MMP-9), all processes with relevance for the pathogenesis of MS.

## Materials and Methods

### Ethic statement

The study was approved by the Regional Committee for Medical and Health Research Ethics in Western Norway. All participants gave written informed consent.

### Study population and design

The OFAMS study was a randomized double-blind, placebo-controlled trial that included 92 Norwegian patients with relapsing remitting (RR) MS according to the McDonald criteria [9]. The inclusion criteria were age 18-55 years, Expanded Disability Status Scale (EDSS) score ≤5, and ≥1 relapse or new T1-weighted gadolinium enhancing (T1Gd^+^) or T2-weighted (T2) MRI lesion the last year. None of the patients used immunomodulatory drugs at inclusion. At baseline the patients were randomized to receive either Triomar™ capsules (Pronova Biocare, Sandefjord, Norway) containing 1350 mg of EPA and 850 mg of DHA or placebo (corn oil). At month six all patients started subcutaneous interferon β-1a (IFNβ) injections thrice weekly. Serum was collected at baseline and after 1, 3, 6, 7, 9, 12, 18 and 24 months. MRI was performed at baseline and monthly during months 1 to 9, and after 12 and 24 months.

### Measurement of inflammation markers

Serum samples were collected by venipuncture and stored at -80°C until analysis. The concentrations of OPG, sTNF-R1, the chemokines CXCL16 and CCL21, TGFβ1, PTX3, MMP-9, IL-1RA, OPN, and ALCAM were measured by enzyme immunoassay (EIA) obtained from R&D systems (Minneapolis, MN, USA). The analyses were performed simultaneously for all samples from each patient. For each marker consecutive samples from each patient were analysed in neighbouring wells on the same plate. The lab technicians were blinded for treatment as well as MRI activity. The intra- and inter-assay coefficient of variation were <10% for all EIAs.

### HLA-DRB1 typing

The HLA-DRB1 status was determined by DNA sequencing using SeCore Loc DRB1 SEQ kit (Invitrogen, Carlsbad, Ca, USA) at the Department of Immunology, Oslo University Hospital, Oslo, Norway.

### MRI measurements

As previously described [9], MRI was performed according to a standardised protocol comprising T2-weighted and T1-weighted Gd^+^ scans using a standard head coil with a 1.5 Tesla MRI Unit. Blinded assessments of T1Gd^+^ lesions, T2 lesions, and combined unique activity (CUA; the sum of T1Gd^+^ lesions and new or enlarging T2 lesions) were conducted by two experienced neuroradiologists.

### Missing values

Due to restricted amount of serum, not all inflammation markers could be measured at all time points. All inflammation markers were, however, measured in at least three serum samples from each patient, except three patients with none or one measurements of IL-1Ra or ALCAM. Fourteen (2.7%) and nine (2.0%) MRI scans were missing during study months 1-6 and 7-24, respectively. The number of patients, MRI scans and measurements of inflammatory markers contributing to each analysis are provided in the results. Body mass index (BMI) was missing for two patients. Missing values were not replaced.

### Statistical analyses

Mean values with standard deviations (SD) are used to describe clinical data and the inflammation markers. The MRI outcomes were skewed towards none or one lesion and were therefore dichotomised as present or absent. Since most inflammation markers were skewed, logarithmically transformed values were used in the statistical analyses. There was a considerable intra-individual variation in logarithmically transformed inflammation markers as measured by the intra-class correlation coefficient (ICC). The association between the inflammation markers and simultaneous or subsequent (one study month later) MRI activity was therefore assessed by a logistic regression model for hierarchical data. The SAS GLIMMIX procedure was used to fit a model with random intercepts for patients and fixed effects for inflammation markers. Only paired measurements of inflammation markers and MRI outcomes were included in the regression model. Due to logarithmic transformation of inflammation markers, odds ratios (OR) for MRI activity associated with each 1 SD increase in inflammation markers were estimated. The impact of gender, age, BMI, and HLA-DRB1*15 status on MRI activity and inflammation markers was assessed by t-test for regression coefficients in a multivariate regression model. Some logarithmically transformed inflammation markers were different in the ω-3 FA and the placebo group at baseline. The consecutive values were therefore divided by corresponding baseline measurements (all on logarithmic scale) before assessing the effect of ω-3 FA treatment. Consequently, this effect could not be examined in patients lacking baseline value for the particular marker. Because of substantial intra-individual variation, the linear mixed model approach was chosen in favour of independent samples t-test for assessing the mean differences in inflammation markers between the ω-3 FA-treated and placebo-treated groups. The homoscedasticity in inflammation markers at different study months was assessed by Levene’s test (SAS GLM procedure). The linear model with random intercepts for patients and indicator variable identifying group-belonging as fixed effect was fitted (SAS MIXED procedure). The adjustment for heteroscedastic variances was introduced into the linear mixed models for inflammation markers, where Levene’s test was significant (highlighted by an asterisk in the table reporting the results). The statistical analyses were conducted using SPSS version 18.0 and SAS version 9.2. Due to multiple testing, Bonferroni correction was performed for the association between MRI activity and inflammation markers during the whole study period, which was considered the main analyses. Assuming 30 tests (10 inflammation markers and three MRI outcomes), p values < 0.0017 were considered significant in this analysis.

## Results

Of the 92 patients included in the OFAMS study, sufficient serum or MRI scans were missing from seven. Thus, 85 patients, 55 (65%) women and 30 (35%) men, age 38.7 (8.1) years, BMI 25.8 (4.3), disease duration 1.9 (3.1) years, and EDSS score at baseline 1.9 (0.8) were included in this study. Mean levels of the inflammation markers at baseline and during different phases of the study are shown in Table 1. The concentrations and the proportion of positive MRI scans at each time point throughout the study are given in Table S1. The ICC for log-transformed inflammation markers varied between 0.43 (MMP-9) and 0.77 (CXCL16), implying that between 23% and 57% of the total variation was intra-individual. The mean concentration of most of the inflammation markers before IFNβ treatment differed from that recorded during IFNβ treatment (Table 1). Gender, age, BMI, or the presence of HLA-DRB1*15 were not associated with any of the markers.

**Table 1:** Concentrations and intra-class correlation coefficients (ICC) of inflammation markers.

**Inflammation marker**	**Baseline**		**Whole study period**			**Omega-3**		**Placebo**		**No IFB**		**IFB**		**p-value^5^**
	**N^1^**	**Mean (SD**)	**n^3^**	**Mean (SD^4^**)	**ICC^2^**	**n^3^**	**Mean (SD^4^**)	**n^3^**	**Mean (SD^4^**)	**n^3^**	**Mean (SD^4^**)	**n^3^**	**Mean (SD^4^**)	
PTX3 (ng/ml)	80	1255	725	1164	67.9	376	1186	349	1141	327	1143	398	1182	<0.001
		(1150)		(723)			(811)		(614)		(852)		(596)	
sTNF-R1 (pg/ml)	81	920	733	980	72.8	384	981	349	979	331	919	402	1030	<0.001
		(254)		(272)			(276)		(268)		(256)		(274)	
CXCL16 (pg/ml)	81	1262	728	1328	76.6	379	1199	349	1468	331	1218	397	1419	<0.001
		(772)		(634)			(379)		(804)		(662)		(595)	
MMP-9 (pg/ml)	81	836	734	585	43.4	384	481	350	700	331	728	403	468	<0.001*
		(1239)		(807)			(404)		(1078)		(937)		(660)	
CCL21 (pg/ml)	80	291	734	328	70.9	384	298	350	361	330	299	404	352	<0.001
		(139)		(161)			(127)		(186)		(142)		(172)	
IL-1Ra (pg/ml)	77	134	718	183	76.3	370	113	348	258	324	130	394	227	<0.001
		(350)		(629)			(230)		(866)		(393)		(768)	
OPN (ng/ml)	73	7.6	724	9.9	53.2	381	10.4	343	9.3	322	7.9	402	11.4	<0.001*
		(3.9)		(6.0)			(6.2)		(5.7)		(4.9)		(6.3)	
OPG (pg/ml)	73	1052	723	1141	60.7	379	1212	344	1062	322	1050	401	1214	<0.001
		(360)		(413)			(490)		(288)		(425)		(389)	
TGFβ1 (ng/ml)	71	21.4	722	20	45.9	379	21.0	343	19.3	320	20.7	402	19.8	0.062
		(8.1)		(7)			(8.1)		(6.4)		(7.5)		(7.3)	
ALCAM (ng/ml)	69	160	683	171	56.8	357	163	326	179	310	170	373	171	0.439
		(81)		(74)			(69)		(78)		(74)		(74)	

^1^ Number of patients with a baseline measurement; ^2^ ICC (%) is calculated for logarithmically transformed data with baseline measurements included; ^3^ Number of measurements in the analyses; ^4^ Calculated ignoring clustering; ^5^ p-value from the model from mixed model with random intercepts and heteroscedastic residuals where relevant (irrelevant indicated with *) for logarithmically transformed measurements

### The association between inflammatory markers and disease activity as assessed by MRI

**Figure 1:**
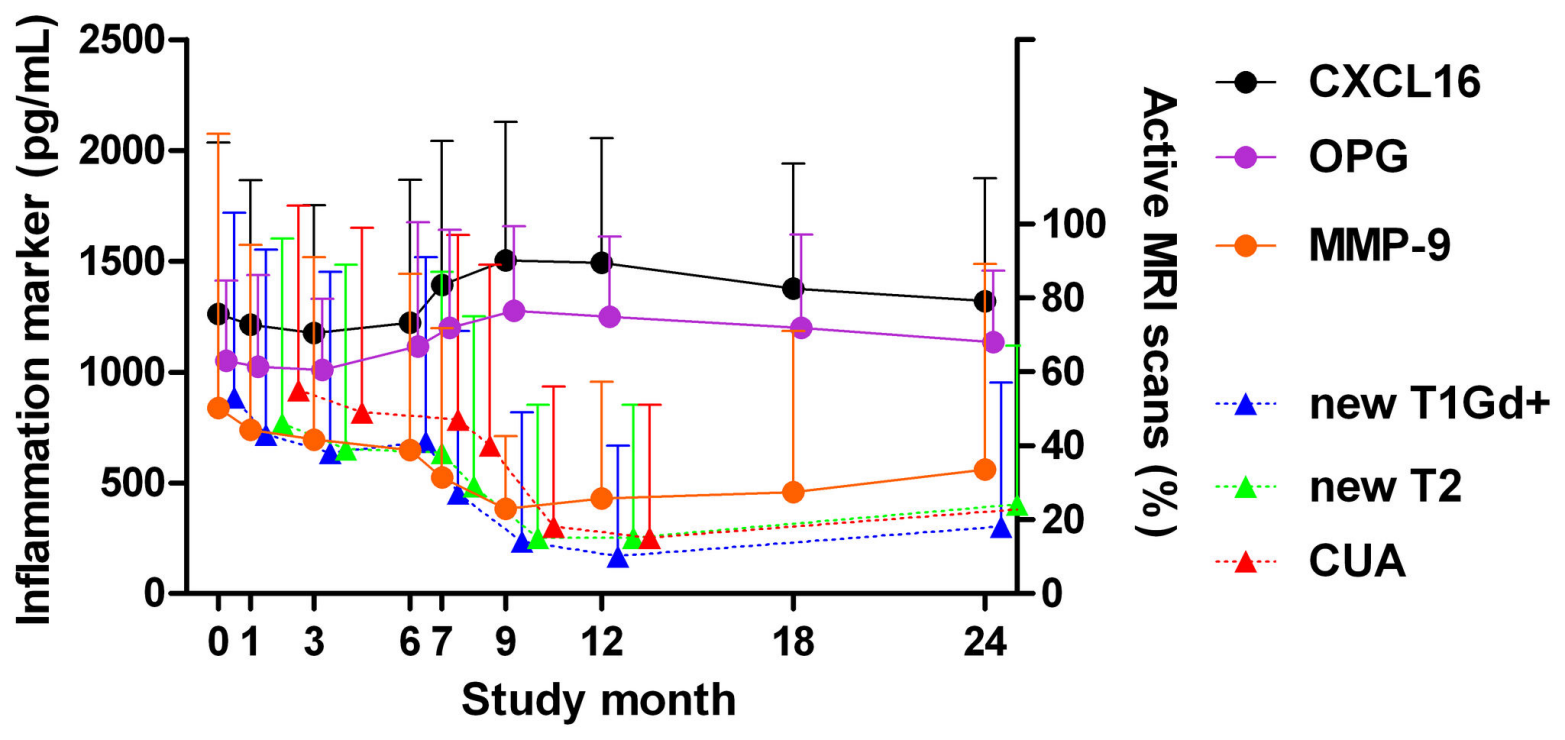
MRI activity and mean concentrations (positive SD) of inflammation markers significantly associated with MRI outcomes throughout the study. All available observations at each time point are included (see Table S1 for numbers). All T1Gd^+^ lesions are shown at month 0 (baseline). Abbreviations: CXCL16: Chemokine (C-X-C motif) ligand 16, OPG: osteoprotegerin, MMP-9: matrix metalloproteinase 9, T1Gd^+^: gadolinium enhancing T1 MRI lesion, CUA: combined unique activity.

To assess whether the inflammation markers reflect disease activity, a logistic regression model for hierarchical data was fitted to estimate the OR for simultaneous MRI activity associated with 1 SD increase in each inflammation marker (Table 2). After Bonferroni correction, increasing concentrations of OPG were associated with lower OR for new T1Gd^+^ and T2 lesions and CUA (i.e., the combination of the two other MR parameters), and increasing levels of CXCL16 were associated with lower OR for new T1Gd^+^ lesions and CUA. In contrast to the inverse associations with these markers, increasing concentrations of MMP-9 were associated with more new T1Gd^+^ lesions. The temporal relationship between MRI activity and CXCL16, OPG and MMP-9 is shown in Figure 1. Less pronounced inverse associations were recorded between MRI activity and IL-1Ra and sTNFR1, and positive associations with TGFβ, but these associations were not significant after Bonferroni correction. None of the associations were influenced by adjusting for gender, age, BMI, or the presence of HLA-DRB1*15 (data not shown).

**Table 2:** Odds ratios for simultaneous MRI activity for each 1 SD increase in each inflammation marker.

	Odds ratio (95% CI)			
	p-value			
Inflammation marker	N	Combined unique activity	New T2 lesions	New T1Gd+ lesions
	(n)			
PTX3^1^	558	1.15 (0.90, 1.49)	1.17 (0.91, 1.49)	1.00 (0.78, 1.30)
	(638)	0.25	0.22	0.98
sTNF-R1^1^	564	0.68 (0.53, 0.88)	0.75 (0.59, 0.96)	0.69 (0.54, 0.89)
	(645)	0.003	0.02	0.005
CXCL16^1^	560	0.56 (0.42, 0.74)	0.66 (0.51, 0.86)	0.48 (0.35, 0.66)
	(641)	<0.001^3^	0.002	<0.001^3^
MMP-9^1^	565	1.37 (1.08, 1.73)	1.32 (1.04, 1.67)	1.50 (1.17, 1.91)
	(646)	0.009	0.02	0.001^3^
CCL21^1^	566	0.83 (0.64, 1.06)	0.92 (0.72, 1.17)	0.74 (0.58, 0.95)
	(646)	0.13	0.49	0.02
IL-1Ra ^2^	555	0.77 (0.60, 0.99)	0.80 (0.62, 1.02)	0.70 (0.53, 0.91)
	(632)	0.04	0.07	0.007
OPN ^2^	563	0.89 (0.72, 1.12)	0.93 (0.74, 1.16)	0.82 (0.65, 1.03)
	(636)	0.323	0.507	0.084
OPG^2^	562	0.56 (0.43, 0.72)	0.61 (0.48, 0.79)	0.55 (0.42, 0.72)
	(635)	<0.001^3^	<0.001^3^	<0.001^3^
TGFβ^2^	563	1.27 (0.99, 1.62)	1.16 (0.92, 1.47)	1.39 (1.07, 1.79)
	(634)	0.05	0.21	0.01
ALCAM^2^	535	0.98 (0.77, 1.25)	1.03 (0.81, 1.31)	1.05 (0.82, 1.34)
	(604)	0.85	0.82	0.70

N: Number of measurements included in the analysis of new T2 lesions and combined unique activity; (n): Number of measurements included in the analysis of new T1Gd+ lesions.

^1^ Analysed in 85 patients. ^2^ Analysed in 84 patients. ^3^ Significant after Bonferroni correction.

As shown in Table 1 and Figure 1, the MRI outcomes and also most inflammation markers differed between before and during IFNβ treatment. OR for simultaneous MRI activity associated with each inflammation marker before (month 0-6) and during (month 7-24) IFNβ treatment is shown in Table 3. A clear pattern was observed for OPG which was inversely associated with all MRI outcomes during IFNβ treatment, whereas no associations were recorded before IFNβ treatment. PTX3 was positively associated with new T2 lesions and CUA before, but not during IFNβ treatment.

**Table 3:** Odds ratios for simultaneous MRI activity for each 1 SD increase in each inflammation marker before and during interferon beta treatment.

Inflammation marker	Before interferon beta				During interferon beta			
	Odds ratio (95% CI)				Odds ratio (95% CI)			
	p-value				p-value			
	N^1^ (N^2^)	Combined unique activity	New NT2 lesions	New T1Gd+ lesions	N^3^ (n)	Combined unique activity	New NT2 lesions	New T1Gd+ lesions
	(n)							
PTX3	242 (322)	1.48 (1.04, 2.13)	1.49 (1.08, 2.07)	1.16 (0.84, 1.59)	316 (85)	1.13 (0.80, 1.59)	1.03 (0.71, 1.49)	1.06 (0.71, 1.59)
	(84)	0.03	0.02	0.38		0.50	0.86	0.79
sTNF-R1	245 (326)	0.92 (0.64, 1.32)	1.01 (0.73, 1.40)	1.02 (0.71, 1.45)	319 (85)	0.75 (0.54, 1.04)	0.72 (0.50, 1.93)	0.70 (0.48, 1.02)
	(85)	0.64	0.95	0.93		0.08	0.07	0.07
CXCL16	245 (326)	0.88 (0.60, 1.28)	0.92 (0.67, 1.28)	0.75 (0.51, 1.10)	315 (84)	0.64 (0.45, 0.93)	0.63 (0.42, 0.93)	0.73 (0.48, 1.11)
	(85)	0.49	0.63	0.15		0.02	0.02	0.14
MMP-9	245 (326)	1.21 (0.84, 1.76)	1.23 (0.88, 1.73)	1.31 (0.91, 1.89)	320 (85)	1.02 (0.75, 1.39)	1.04 (0.75, 1.44)	1.03 (0.72, 1.47)
	(85)	0.31	0.22	0.15		0.90	0.81	0.87
CCL21	245 (325)	0.99 (0.68, 1.45)	1.13 (0.80, 1.59)	0.92 (0.66, 1.30)	321 (85)	0.98 (0.71, 1.36)	0.98 (0.69, 1.39)	0.94 (0.65, 1.35)
	(85)	0.98	0.50	0.65		0.90	0.92	0.72
IL-1Ra	242 (319)	0.96 (0.67, 1.39)	0.99 (0.71, 1.38)	1.02 (0.70, 1.47)	313 (84)	0.99 (0.72, 1.38)	0.95 (0.67, 1.35)	0.93 (0.64, 1.35)
	(84)	0.81	0.94	0.93		0.98	0.79	0.69
OPN	244 (317)	1.12 (0.79, 1.58)	1.10 (0.80, 1.51)	1.10 (0.78, 1.54)	319 (85)	1.28 (0.91, 1.79)	1.24 (0.87, 1.77)	1.25 (0.85, 1.85)
	(85)	0.51	0.56	0.58		0.16	0.24	0.25
OPG	244 (317)	0.93 (0.66, 1.32)	1.03 (0.75, 1.40)	1.00 (0.72, 1.40)	318 (85)	0.55 (0.38, 0.81)	0.47 (0.31, 0.71)	0.53 (0.35, 0.81)
	(85)	0.69	0.86	0.99		0.002	<0.001	0.004
TGFβ1	244 (315)	1.31 (0.92, 1.88)	1.18 (0.86, 1.63)	1.39 (0.96, 2.03)	319 (85)	1.10 (0.80, 1.51)	1.06 (0.76, 1.47)	1.04 (0.73, 1.48)
	(85)	0.14	0.29	0.08		0.57	0.75	0.87
ALCAM	236 (305)	1.04 (0.72, 1.49)	1.10 (0.80, 1.53)	1.20 (0.86, 1.66)	299 (81)	0.89 (0.64, 1.22)	0.90 (0.64, 1.27)	0.89 (0.61, 1.30)
	(82)	0.84	0.55	0.28		0.46	0.55	0.55

N^1^ (N^2^): Number of measurements included in the analysis of new T2 lesions and combined unique activity (new T1Gd+ lesions); N^3^: Number of measurements included in the analysis; (n): Number of patients included in the analysis.

To assess whether markers of systemic inflammation also predict disease activity, we estimated the relationship between 1 SD increase in each marker and MRI activity one month later (Table 4). The association between CXCL16 and subsequent CUA was equal to that recorded for simultaneous CUA, whereas the ORs for new T1Gd^+^ and T2 lesions were somewhat higher but in the same order of magnitude as for simultaneous disease activity. Except a weak association between OPG and new T1Gd^+^ lesions the next month, none of the other inflammation markers were associated with MRI activity the following month. None of the associations with subsequent MRI activity reached a significance level of 0.0017, although the association between CXCL16 and CUA was close (p = 0.0021).

**Table 4:** Odds ratio for subsequent MRI activity for 1 SD increase in each inflammation marker (MRI outcomes lagged by one month).

		Odds ratio (95% CI) p-value		
Inflammation marker		Combined unique activity	New T2 lesions	New T1Gd+ lesions
	N			
PTX3^1^	393	1.09 (0.81, 1.47)	1.05 (0.80, 1.39)	1.01 (0.75, 1.36)
		0.57	0.73	0.96
sTNF-R1^1^	396	0.79 (0.58, 1.09)	0.81 (0.61, 1.09)	0.78 (0.56, 1.07)
		0.15	0.17	0.12
CXCL16^1^	395	0.65 (0.46, 0.93)	0.60 (0.43, 0.83)	0.58 (0.40, 0.84)
		0.02	0.002	0.004
MMP-9^1^	396	1.17 (0.87, 1.58)	1.20 (0.90, 1.60)	1.14 (0.84, 1.54)
		0.31	0.21	0.41
CCL21^1^	395	1.03 (0.75, 1.41)	0.94 (0.71, 1.26)	0.83 (0.61, 1.13)
		0.85	0.70	0.23
IL-1Ra^2^	389	0.85 (0.62, 1.17)	0.86 (0.64, 1.15)	0.88 (0.64, 1.21)
		0.32	0.30	0.42
OPN^1^	388	0.91 (0.68, 1.21)	0.88 (0.69, 1.16)	0.93 (0.69, 1.25)
		0.51	0.37	0.61
OPG^1^	387	0.78 (0.57, 1.06)	0.85 (0.63, 1.13)	0.68 (0.49, 0.95)
		0.11	0.25	0.02
TGFβ1^1^	386	1.40 (1.00, 1.96)	1.17 (0.87, 1.58)	1.20 (0.86, 1.67)
		0.05	0.31	0.28
ALCAM ^3^	369	0.90 (0.66, 1.23)	0.87 (0.66, 1.17)	0.86 (0.63, 1.18)
		0.50	0.36	0.34

N: Number of measurements included in the analysis of new T2 lesions and combined unique activity (n): Number of measurements included in the analysis of new T1Gd+ lesions.

^1^ Analysed in 85 patients. ^2^ Analysed in 84 patients. ^3^ Analysed in 82 patients.

### The effect of ω-3 FA treatment on inflammatory markers

We have recently reported that the treatment with EPA and DHA had no influence on disease MRI activity in these patients [9]. The effect of ω-3 FA treatment on the inflammation markers is shown in Table 5. As compared with placebo, ω-3 FA treatment did not induce any significant change in any of the inflammation markers before or after initiation of IFNβ, or during the whole study period.

**Table 5:** The effect of treatment with ω-3 fatty acids versus placebo on inflammation markers.

Inflammation marker		Before interferon beta		During interferon beta		Whole study period	
		(Month 1-6)		(Month 7-24)		(Month 1-24)	
	N	N_1_, N_2_^1^	Mean difference^3^	N_1_, N_2_^2^	Mean difference^3^	N_1_, N_2_^1^	Mean difference^3^
			(p-value^2^)		(p-value^2^)		(p-value^2^)
PTX3 (pg/ml)	80	128,107	75.0 (0.58)	200,175	-31.8 (0.83)*	328, 282	8.6 (0.93)*
sTNF-R1 (pg/ml)	81	131,107	21.2 (0.95)	207,175	15.9 (0.89)*	340, 282	17.3 (0.92)*
CXCL16 (pg/ml)	81	131,107	-267.6 (0.14)	204,175	-273.4 (0.61)*	335, 282	-273.6 (0.30)*
MMP-9 (ng/ml)	81	128,11	-275.1 (0.57)	205,18	-178.7 (0.20)	333, 290	-214.3 (0.33)*
CCL21 (pg/ml)	80	125,11	-65.4 (0.62)	203,18	-80.3 (0.39)	328, 290	-74.7 (0.42)*
IL-1Ra (pg/ml)	77	122,104	-93.0 (0.91)*	191,17	-227.6 (0.42)*	313, 274	-176.9 (0.50)*
OPN (ng/ml)	73	122,92	1.3 (0.08)*	196,152	1.5 (0.14)	318, 244	1.4 (0.13)*
OPG (pg/ml)	73	121,93	154.0 (0.86)*	195,152	151.2 (0.84)*	316, 245	151.7 (0.85)*
TGFβ1 (ng/ml)	71	118,91	2.6 (0.58)*	191,149	1.0 (0.91)	309, 240	1.6 (0.81)
ALCAM (ng/ml)	69	116,88	-13.9 (0.39)*	176,144	-14.4 (0.48)	292, 232	-14.1 (0.37)*

N: number of patients in the analysis.

^1^ N_1_ and N_2_ stand for sample size in ω-3 and placebo group, respectively; ^2^ p-value from the model from linear mixed model with random intercepts (* models with adjustment for heteroscedastic residuals in different study months incorporated) for logarithmically transformed and baseline-adjusted measurements; ^3^ Mean difference calculated on original scale with baseline measurements excluded.

## Discussion

We have tested a broad panel of inflammation markers, of which CXCL16 and OPG were inversely, and MMP-9 positively associated with simultaneous MRI activity. Treatment with EPA and DHA did not alter the serum levels of any of these inflammation markers.

Several chemokines and chemokine receptors, including CXCL8, CXCL13, CXCL12, CCL2, and CCL19 have been suggested as possible biomarkers in MS [10–13]. In this study CXCL16 stood out compared to the other markers as it reflected simultaneous MRI activity and was also associated with a trend for low subsequent MRI activity. CXCL16 is expressed on the surface of antigen presenting cells where it serves as a ligand for CXCR6 on T cells and natural killer T cells. The expression of CXCL16 is induced by inflammatory mediators including IFNγ and TNFα, and it can be shed into the circulation as a soluble chemokine that activates CXCR6 expressing cells [14]. Both CXCL16 and CXCR6 are expressed in the CNS, and protect against excitotoxic damage caused by excessive glutamate exposure and oxygen and glucose deprivation [15]. CXCL16 has also been shown to regulate T cell homing to the CNS in experimental autoimmune encephalomyelitis (EAE) [16], and it was recently shown in this model that immature myeloid cells expressing CXCL16 redirect CXCR3 ^+^ CXCR6^+^ and myelin-specific T cells from CNS to lymph nodes [17]. To our knowledge, only one cross-sectional study has previously analyzed CXCL16 levels in MS patients, reporting higher levels in CSF compared to serum and higher levels in patients with MS and other inflammatory CNS diseases compared to controls with non-inflammatory neurological diseases [18]. Very recently CXCL16 was shown to be expressed by macrophages and astrocytes in MS lesions [19]. Our findings call for further studies in larger MS populations with longer follow-up.

OPG and sTNF-R1 are members of the TNF receptor family, while IL-1Ra is a receptor antagonist. These soluble receptors and receptor antagonists may attenuate the pro-inflammatory activity of their respective ligands, i.e., receptor activator of nuclear factor kappaB ligand (RANKL), TNFα, and IL-1. Injection of sTNF-R1 prevents EAE [20], whereas the role in MS is less clear. IL-1Ra has been linked to MRI activity in MS patients [21], but the study was too small to reach a firm conclusion. Previous small studies have reported higher serum OPG levels in MS patients compared to healthy controls [22], and no difference in CSF OPG levels between patients with MS and patients with non-inflammatory neurological diseases [23]. High serum levels of sTNF-R1 have been reported during [24] and after [25] relapses in longitudinal studies, suggesting a possible protective role in MS patients. A recent cross sectional study was however negative [26]. Our findings that circulating levels of OPG were inversely associated with MRI activity, and the corresponding trends for sTNF-R1 and IL-1Ra, may suggest a beneficial effects of these cytokine modulators in MS, possibly mediated through their ability to block their corresponding pathogenic ligands.

Unlike most of the examined markers, MMP-9 was positively associated with MRI activity. MMP-9 is secreted by activated T cells and microglia, and is involved in the digestion of extracellular matrix and loss of blood brain barrier (BBB) integrity [27]. Thus the observed association with new T1Gd+ lesions fits well since such MRI activity is a marker of BBB disruption. High levels of MMP-9 have previously been reported to predict [28], and in agreement with our results reflect [29], MRI activity in MS. A similar trend, although not significant after Bonferroni correction, was recorded for TGFβ1. It is believed that the main role of TGFβ1 is to maintain immune tolerance and that it protects against inflammatory demyelination [30,31]. However, recent studies suggest that TGFβ1 could induce the generation of autoreactive Th17 cells [32], a subset believed to promote disease activity in MS. In addition, TGFβ1 signaling in the CNS has been shown to precede paralysis in EAE, independent of Th17 cells [33]. Further studies are needed to clarify the role of this pluripotent cytokine in MS.

For several markers, including CXCL16 and MMP-9, analyzing the periods before and during IFNβ treatment yielded weaker associations than those recorded for the whole study period. This is compatible with the well known effect of IFNβ on inflammation and MRI activity in MS, and the marked drop in MRI activity and alteration in the concentrations of inflammation markers between month 6 and 7 recorded in this study. It is therefore likely that IFNβ had a substantial influence on our results. However, as all patients received IFNβ from month 6, we cannot draw definite conclusions on the effect of this treatment. PTX3 was associated with high OR for MRI activity before, but not during IFNβ treatment. Although this observation concurs with a recent report on the association between PTX3 and clinical disease activity [34], it may be an incidental finding.

Our findings corroborate the inability of EPA and DHA to modulate disease activity in MS [9]. Previous studies on the effect of EPA and DHA on inflammatory markers in MS have reached conflicting results. Gallai et al. reported a significant decrease in the production of IL-1β, TNFα, IL-2, and IFN-γ in peripheral blood mononuclear cells (PBMC) upon daily supplementation with 3.0 g of EPA and 1.8 g of DHA [35], whereas Weinstock-Guttman et al. found no effect on plasma levels of a range of inflammatory markers of 1.98 g EPA and 1.32 g DHA compared to 6 g olive oil per day [36]. EPA and DHA have also been demonstrated to reduce MMP-9 synthesis in PBMC from MS patients [37]. Diverging results across studies may be due to differences in doses, measurements in PBMC versus serum/plasma, and in the content of the placebo preparations [7]. The corn oil capsules used as placebo in the OFAMS study contained 52% linoleic acid. Although most studies have shown no effect of linoleic acid on systemic inflammation markers [38], we cannot exclude an effect on some of the inflammation markers.

The prospective design with repeated measurements of MRI and inflammation markers in each patient allowed us to capture both intra- and individual variation, and is a strength of this study. The finding that up to 57% of the total variation of log transformed levels of the inflammation markers was inter-individual underscores the advantage of this approach. Moreover, MRI scanning, collection of serum samples and measurement of inflammation markers were standardized in all patients. There are, however, also limitations to this study. The restricted amount of serum did not allow measurement of all inflammation markers in all patients, and the number of patients may have been too low to detect minor effects of ω-3 fatty acids on disease activity. Although IFNβ was always injected in the evening and serum was always sampled in the morning, the interval varied with up to 48 hours. This could have affected the concentrations of some of the inflammation markers that respond rapidly to IFNβ, and increased the variability in the latter phase of the study. Any such effect would, however, affect patients on ω-3FA and placebo equally. Moreover, the SD of the inflammation markers was in the same order of magnitude before and during IFNβ treatment, arguing against any substantial effect. Another potential weakness is that the interval between MRI scannings was markedly longer at the end of the study. The MRI scans were distributed to capture the high expected disease activity prior to IFNβ treatment, and this could potentially influence the ratio between new T1Gd+ lesions and new T2 lesions during different phases of the study. There was, however, an even reduction in new T1Gd^+^ and T2 lesions after the initiation of IFNβ, and this effect is therefore likely to be minor.

## Conclusions

In this longitudinal study serum levels of CXCL16, OPG and MMP-9 reflect MRI activity in RRMS, suggesting that these markers, in particular CXCL16 which also may predict disease activity, could be potential markers of disease activity in MS. Treatment with EPA and DHA did not affect the serum concentrations of these or other inflammation markers. The results obtained in this exploratory study need to be confirmed and should encourage further studies, particularly on CXCL16 as a potential mediator and biomarker in MS.

## Supporting Information

Table S1 **Concentrations of inflammation markers and frequencies of positive MRI scans throughout the study.**
(DOCX)
