# Supplementary material for: Inflammation Markers in Multiple Sclerosis: CXCL16 Reflects and May Also Predict Disease Activity
Source: PLoS One. 2013 Sep 19;8(9):e75021. doi: 10.1371/journal.pone.0075021 (PMC3777920; doi:10.1371/journal.pone.0075021)
Supplement: Table S1 — Concentrations of inflammation markers and frequencies of positive MRI scans throughout the study. (DOCX) [file pone.0075021.s001.docx]

Table SI. Concentrations of inflammation markers and frequencies (%) of positive MRI scans (new T2 lesions, new T1Gd+ lesions and combined unique activity) at different time points.

|  | Study month | | | | | | | | |
| --- | --- | --- | --- | --- | --- | --- | --- | --- | --- |
|  | 0 | 1 | 3 | 6 | 7 | 9 | 12 | 18 | 24 |
| Inflammation marker | N  Mean (SD) | N  Mean (SD) | N  Mean (SD) | N  Mean (SD) | N  Mean (SD) | N  Mean (SD) | N  Mean (SD) | N  Mean (SD) | N  Mean (SD) |
| PTX3  sTNF-R1  CXCL16  MMP-9  CCL21  IL-1Ra  OPN  OPG  TGFβ1  ALCAM | 80  1255 (1150)  81  920 (254)  81  1262 (772)  81  836 (1239)  80  291 (139)  77  134 (350)  73  7.6 (3.9)  73  1052 (360)  71  21.4 (8.1)  69  160 (81) | 81  1129 (919)  82  927 (279)  82  1213 (652)  82  739 (835)  82  301 (147)  81  121 (365)  82  8.0 (4.7)  82  1025 (411)  82  20.5 (7.8)  79  167 (68) | 83  1077 (560)  84  914 (243)  84  1177 (577)  84  695 (822)  84  292 (126)  83  94 (212)  84  8.2 (5.9)  84  1009 (321)  84  19.7 (7.0)  81  178 (79) | 83  1116 (674)  84  915 (253)  84  1222 (646)  84  646 (796)  84  312 (155)  83  169 (563)  83  7.9 (4.8)  83  1115 (561)  83  21.4 (7.3)  81  172 (67) | 83  1277 (682)  83  1035 (286)  82  1393 (650)  83  523 (673)  83  334 (172)  82  244 (777)  83  11.6 (6.2)  82  1200 (442)  83  19.9 (8.0)  76  160 (68) | 81  1215 (623)  82  1072 (291)  81  1504 (625)  82  382 (328)  82  361 (176)  80  273 (963)  82  11.2 (6.7)  82  1277 (380)  82  19.6 (7.5)  77  175 (79) | 83  1181 (561)  84  1056 (274)  83  1492 (563)  84  428 (528)  85  373 (166)  83  234 (796)  84  11.5 (6.1)  84  1249 (362)  84  20.0 (6.9)  77  178 (70.2) | 78  1068 (518)  79  999 (246)  78  1375 (565)  79  457 (727)  79  351 (173)  77  192 (641)  79  11.6 (6.1)  79  1199 (420)  79  19.3 (7.3)  72  169 (69) | 73  1159 (572)  74  983 (268)  73  1319 (555)  75  560 (926)  75  338 (173)  72  186 (605)  74  11.3 (6.6)  74  1135 (322)  74  20.0 (6.7)  71  176 (83) |
| MRI outcomes | N (%) | N (%) | N (%) | N (%) | N (%) | N (%) | N (%) | N (%) | N (%) |
| combined unique activity  New T 2 lesions  New T1 Gd+ lesions | 0  0  45 (52.9) | 46 (55.4)  36 (43.4)  38 (45.8) | 40 (48.8)  31 (37.8)  32 (39.0) | 40 (47.1)  35 (41.2)  32 (37.6) | 33 (39.8)  22 (26.5)  24 (28.9) | 15 (17.6)  12 (14.1)  13 (15.3) | 13 (15.5)  8 (9.5)  13 (15.5) | 0  0  0 | 19 (23.2)  15 (18.3)  20 (24.4) |
